# Use of Radiofrequency Ablation in Benign Thyroid Nodules: A Literature Review and Updates

**DOI:** 10.1155/2013/428363

**Published:** 2013-11-03

**Authors:** Kai-Pun Wong, Brian Hung-Hin Lang

**Affiliations:** Division of Endocrine Surgery, Department of Surgery, The University of Hong Kong, Queen Mary Hospital, Pokfulam Road, Hong Kong

## Abstract

Successful thermal ablation using radiofrequency has been reported in various tumors including liver or kidney tumors. Nonsurgical minimally invasive ablative therapy such as radiofrequency ablation (RFA) has been reported to be a safe and efficient treatment option in managing symptomatic cold thyroid nodules or hyperfunctioning thyroid nodules. Pressure and cosmetic symptoms have been shown to be significantly improved both in the short and long terms after RFA. For hyperfunctioning thyroid nodules, RFA is indicated for whom surgery or radioiodine are not indicated or ineffective or for those who refuse surgery or radio-iodine. Improvement of thyroid function with decreased need for antithyroid medications has been reported. Complication rate is relatively low. By reviewing the current literature, we reported its efficacy and complications and compared the efficacy of RFA relative to other ablative options such as ethanol ablation and laser ablation.

## 1. Introduction

Thyroid nodules are commonly found and they pose one of the most common clinical problems for primary physicians and specialists. Clinically palpable nodules are found in 5–10% of the normal population and nonclinically palpable nodules such as those found incidentally on ultrasound (US) occur in up to 67% [1–3]. With the increasing use of US, it is expected that more and more asymptomatic thyroid nodules would be detected [4].

Although the majority of these nodules are benign and may not cause symptoms, some are occasionally associated with pressure or thyrotoxic symptoms [5, 6]. Surgery is the treatment of choice for these nodules but may be associated with certain operative morbidity, unsightful scar and permanent hypothyroidism [7–10]. The alternatives include ethanol ablation (EA) [11–13], radiofrequency ablation (RFA) [5, 11, 14–24], percutaneous laser ablation (PLA) [6, 25], high-intensity focused ultrasound (HIFU) [26], and microwave ablation [27, 28]. Nonsurgical, minimally invasive modalities, like ethanol ablation and laser ablation, have been reported to be an effective option in treating thyroid nodules [12, 13, 25, 29–33]. RFA is a safe percutaneous ablative technique in treating liver tumors [34, 35]. Since the first reported series in 2006 [36], there had been numerous studies showing its efficacy and safety in treating benign cold and hyperfunctioning thyroid nodules [5, 11, 14–24]. In this paper, we described the basic principles, patient selection, procedure, efficacy, and complications of RFA and its efficacy relative to other ablation modalities.

## 2. Basic Principles of RFA

RFA refers to hyperthermic ablation by high frequency alternating electric current oscillating between 200 and 1200 kHz [37]. By applying an electrode into nodule, a high-frequency alternating current is generated and passed into the surrounding tissue. It thus induces rapid vibration of surrounding ions and frictional heat. Coagulative necrosis and irreversible damage is induced near the electrode at temperature between 50°C and 100°C [38]. Temperature higher than 100°C would lead to vaporization and carbonization which result in increased impedance of the tissue and thus interrupted the transferal of electric current and heat energy [38]. Beside the frictional heat produced by oscillating ions, conduction of heat causes further damages to the surrounding remote area in a slower manner [37, 39]. This forms the basic principles of RFA.

The principles of heat conduction have been demonstrated in one recent experimental study using the pig animal model [40]. In this experimental study, temperature measurements around the RFA electrode in the porcine thyroid gland were taken. The maximum temperature at a distance of 5 mm from the RFA electrode was between 44°C and 61°C while, at a distance of 10 mm, a maximum temperature of 53°C was achieved. Even at this temperature, there were signs of irreversible cell death damage in the region of the thermal lesions [40].

## 3. Patient Selection

In 2012, the Korean Society of Thyroid Radiology made a consensus statement regarding the treatment of thyroid nodules with RFA [41]. Essentially, RFA is indicated either for patients with nodule-related symptoms or with hyperfunctioning nodule(s) which is causing thyrotoxicosis. It is important that, before the ablation, the nodule should be confirmed to be benign in nature with at least two separate US-guided fine-needle aspiration cytologies and/or core biopsies [42, 43]. Report of RFA incompletely treating primary thyroid carcinoma was present and histological report reviewed the inadequacy of RFA in treating primary thyroid carcinoma [44], and there was no evidence in supporting the treatment benefit of RFA in primary thyroid carcinoma. On the other hand, a cytological diagnosis of follicular or indeterminate lesion requires histology to exclude malignancy. Therefore, the consensus statement did not recommend RFA for follicular lesion or a nodule suspicious of malignancy [41]. In general, RFA is a safe procedure. However, we should be cautious in application of RFA in patients who are either pregnant or having history of serious heart problems [5, 41, 45–47]. Since there had been reports of cardiac complication during RFA for liver tumors [45, 48], patients with serious heart disease should have continuous cardiac monitoring during and after RFA of thyroid nodules.

## 4. Procedural Steps

During the procedure, the patient should be positioned in supine with neck slightly extended. Local anesthetic with lignocaine or xilocaine is then injected underneath the skin near the cervical-surrounding soft tissue and thyroid capsule [23, 49, 50]. Some would also administer premedication of fentanyl and midazolam to minimize discomfort [51]. Ground adhesive pads are adhered to both thighs and are connected to RF generator, and the generator was connected to RF electrode.

There are two types of RF device and technique for thyroid nodules. The first technique is called the “fixed ablation” technique. This technique was popularized by an Italian group. It involves the use of a multi-tined expandable electrode [22–24]. The electrode is a 14-gauge, 10 cm long, four-hook expandable needle [14, 22]. Under US guidance, the electrode is inserted along the greatest dimension of the nodule. The hook is then opened to a maximum of 3.5 cm and placed with caution so as to avoid injury to vital structures. Each hook is recommended to be 10 mm away from thyroid capsule, 5~6 mm from pseudocapsule of the outer edge of nodule, and 15 mm from heat-sensitive cervical structures [22]. Lidocaine is injected into superficial cervical tissue and on the thyroid gland capsule under US guidance. Correct position of electrode tips and hook is assessed by US. With this technique, a spherical ablative zone is usually achieved. After the ablation, the hooks are retracted and the electrode is slowly withdrawn after the RF energy has been switched off.

The second technique is called the “moving shot” technique. This technique was first described by Baek et al. in Seoul, Korea. In contrast to the fixed ablation technique, a straight internally-cooled electrode is used [15, 16]. The electrode is usually 15 cm in length and 17 gauge in size with 1 cm active tip [15, 36, 52]. Recent modifications have allowed even shorter (7 cm shaft length) and smaller (18-19 gauge) electrode with active tips around 0.5, 0.7, 1, 1.5, and 2 cm [6, 16]. With these shorter and smaller electrode, it allows better control and variation of ablation option in treating small or vital structure closed thyroid nodule. In the “moving shot technique” [15, 16], the target thyroid nodule is divided into multiple small conceptual ablation units and during the procedure, each conceptual unit is being ablated by the moving ablation electrode tip. The electrode is inserted through the isthmus under the US guidance. As a result, the whole course of electrode could be seen and that greatly reduces the risk of injury of the nearby structures. The ablation first starts from the deepest layer up and so the electrode is slowly withdrawn to the surface. It is important that the region close to the trachea-esophageal groove be underablated in order to avoid injury to the recurrent laryngeal nerve, trachea, and esophagus as this area is often referred to as the “danger triangle”.

## 5. Short- and Long-Term Clinical Efficacy of RFA

RFA therapy has mainly been aimed at decreasing pressure symptoms, improving the cosmetic results as well as resolving thyrotoxic status in hot nodules.  Table 1 shows the results of volume reduction of cold thyroid nodule after RFA [53]. For cold nodules, the efficacy of RFA has mainly been evaluated in terms of reduction of nodule volume, pressure symptoms, and cosmetic symptoms. The reported mean volume reductions at 1- and 6-month were 33~53% and 51~92%, respectively [15, 22]. Most patients have reported improvement in pressure symptoms and cosmetic symptoms [5, 15, 17, 23, 24, 52]. Faggiano et al. reported a prospective study and found that RFA was far superior to conservative treatment [24]. In this study, 20 patients were assigned to the RFA group while the other 20 patients to the control group. After 12 months, patients in the RFA group had significantly decreased mean nodule size (13.3 to 1.8 mL, *P* < 0.001) while, in the control group, the mean nodule size was nearly static (11.2 to 11.8 mL, *P* > 0.05). The symptom score was also significantly improved in the RFA group (3.4 to 0.6 out of 6, *P* < 0.001) and there was a trend of worsening symptoms in the control group (3.0 to 4.1 out of 6, *P* > 0.05). Furthermore, the effect of RFA appeared to be durable. After a 2-year follow-up, a mean of 79.4 ± 2.5% decrease in nodule size (baseline size 24.5 ± 2.1 mL) was observed in an Italian study [23]. Compressive symptoms improved in all patients and were completely resolved in 88% patients [23]. Similarly, Lim et al. reported a high mean nodule volume reduction (93.5 ± 11.7%) after a mean follow up of 49 months. Regrowth of more than 50% was very uncommon (5.6%) [20].

However, it would appear that its efficacy solely depends on the proportion of cystic component within the ablated nodule. For cystic nodules with <10% solid component, RFA could achieve >90% reduction at 6-month after ablation [11, 21, 36]. However, relative to EA, RFA was not superior and required more sessions and was more expensive [11, 21]. In a recent randomized study of single-session treatment of benign cystic thyroid nodules, the mean volume reduction was 96.9% in EA while it was 93.3% in RF ablation (*n* = 21 for each) (difference, 3.6%; lower bound of the one-sided 95% CI of the difference, 1.2%), thus demonstrating the noninferiority of EA to RFA [21]. The authors concluded that EA may be the first-line treatment modality for cystic thyroid nodules, which has comparable therapeutic efficacy to, but is less expensive than, RF ablation [21]. Therefore, EA would still be the first-line ablative measure for cystic nodule. On the other hand, predominant cystic nodule (10–50% solid component) might be suitable for RFA as 6.1–21% failure rates in EA were reported for this type of nodules [19, 54, 55]. RFA is generally good in treating the solid component of these refractory nodules [19, 52]. In predominantly solid or solid nodule (i.e., >50% solid component), RFA could achieve a 23 to 37% volume reduction at the 1st month and 51 to 77% reduction at the 6th month [16, 24, 36]. The rate of volume reduction appears to be maximum after 1–3 months and tends to wean off after 6 months [15, 36]. Besides presence of high cystic content [15–17, 19, 20, 36], low vascularity of nodule [16, 36] and nontoxic status [14] are good predictors for volume reduction.

Table 2 shows the result in patients underwent RFA for hyperfunctioning thyroid nodule. For benign hyperfunctioning thyroid nodules, RFA not only reduces the volume but also improves the functional status. The majority has improved thyroid function and reduced the need for antithyroid medication [22, 24]. In fact, antithyroid medication could be stopped in about 23 to 89% of the patients [16, 22]. In a largest reported series of 28 patients, Spiezia et al. reported that all patients with pretoxic thyroid nodule and 53% of patients with toxic thyroid nodule stopped antithyroid medication at 12-month follow up after RFA. Relative to cold nodules, ablation of hyperfunctioning thyroid nodules achieves lower volume reduction (60% versus 76% at 12 month) [23] and requires more number of sessions (2.2 versus 1.4) [15, 16]. In addition, it is important to be more cautious during ablation because incomplete ablation leading to nodule regrowth and hyperthyroid relapse appeared more common in ablation of hot nodules. Therefore, more sessions of RFA are generally needed [16].

## 6. Complications

Various complications have been described during RFA and they include pain, voice changes, skin burn, hematoma, nodule rupture, and thyroid function disturbance. Most of the patients recover well with proper treatment with very few complications [5, 16–18, 23, 24, 33, 56]. In a Korean multicenter study involving 1459 patients, there were 3.3% patients with complications and, of these, 1.4% had major complications.

Pain is the most common reported complication during the procedure. It occasionally radiates to ear, shoulder, jaw, and chest [6, 16]. However, it is usually self-limiting and resolved soon when the power of RFA has been switched off. It is controlled with simple oral analgesic and only 5.5% of patients require analgesic for more than 2 days [15, 22, 57]. 

Voice change after RFA is uncommon (about 1%) but, nevertheless, it is the most fearful and serious complication [18]. It is likely caused by thermal injury to recurrent laryngeal nerve or sometime vagal nerve in case of large thyroid nodule. Most of the patients recover within 3 months [30]. To reduce this, underablation near tracheoesophageal groove is recommended.

Unlike RFA in liver tumor, there have been no reports of skin burn of thigh pads. This is probably because of the lower energy used during ablation [37]. Skin burn at puncture site has been reported and is usually of first degree. Most patients recover from pain and skin color change within 7 days [18, 36]. Application of ice bag to puncture site might prevent skin burn [18].

Haematoma after thyroidectomy is a distress complication [58]. It happens after RFA but could be managed conservatively with the compression of neck for several minutes [59]. It is usually caused by injury of perithyroidal or anterior jugular vessels during electrode insertion. Proper assessment of perithyroidal and anterior neck and use of small size needle might prevent mechanical injury during insertion [59].

Nodule rupture and thyroid function disturbances are two potential late complications of RFA. Nodule rupture may occur 1 month after RFA. It usually presents as a sudden neck bulging and pain at the time of rupture. It is caused by breakdown of thyroid capsule and internal bleeding. These patients should be managed with antibiotics and closely monitored since abscess formation is a potential sequel requiring subsequent operation [18].

Transient thyrotoxicosis immediately after RFA has been reported. All patients were asymptomatic and spontaneously recovered within 1 month [15, 36]. Though subclinical hypothyroidism in one patient was detected 6 month after RFA, it was inconclusive in causal relationship of RFA and hypothyroidism. Since this patient have elevated antithyroid perioxidase antibody prior RFA, hypothyroidism might be the progression of Hashimoto's thyroiditis [16, 18]. Ha et al. reported that RFA did not affect thyroid function even in patients who had undergone lobectomy [5].

Although there were no fatal complication or ultramajor complication, tracheal injury, esophageal injury, or permanent voice changes have been reported, it is important to be cautious during the procedure and always trace the electrode tip before starting ablation [60]. There has been a complication of brachial plexus injury reported in 1459-patient study [18]. Though rare, to minimize these complications, studying preventive measures and following the consensus guidelines are essential [41].

## 7. Comparison with Other Ablative Treatment

Other than RFA, other minimally invasive ablative modalities have been described, including EA and PLA. EA has been shown to be more effective in treating predominantly cystic nodule (>90% cystic component) [12, 13, 29, 30] and less for predominantly solid nodule [61]. Sung et al. tried to evaluate the opium first-line treatment of thyroid cystic nodule by comparing ethanol ablation and RFA [11, 21]. After a single treatment session, EA achieved similar and noninferior outcome in terms of nodule volume, symptoms, and cosmesis, compared to RFA [21]. However, fewer sessions of ablation were needed in the EA group, and cost of each session of ablation was also less expensive. Therefore, the author concluded that ethanol ablation should be the first-line treatment for cystic nodule. But there was still a role of RFA in ethanol refractory thyroid nodules. About 20% of cystic nodule was refractory to ethanol ablation [12, 19, 54, 55]; additional RFA could effectively treat incompletely resolved thyroid nodule [52]. For predominantly cystic nodules (i.e., >20% solid component), EA is prone to incomplete ablation and adding RFA might be needed for improved results [19].

Since the introduction of PLA in 2000, different studies have assessed its efficacy and safety in the ablation of thyroid nodules [25, 31–33]. It has been recommended by the guidelines of the American Association of Clinical Endocrinologists, the Associazione Medici Endocrinologi (Italian Association of Clinical Endocrinologists), the European Thyroid Association (AACE-AME-ETA) as a possible option for treatment of benign thyroid nodules [57]. PLA could achieve a significantly larger ablation area [40], but current studies have not shown a superiority over RFA [6]. After PLA, comparable volume reduction rate to RFA has been reported [6]. However, durability might not be slightly inferior. Valcavi et al. reported a series of 122 patients undergoing laser ablation of cold thyroid nodule [62]. At 3-year follow-up, mean volume decreased 48 ± 33.1%, while symptoms improved in 73% of patients. For the study of RFA, Spiezia et al. reported a study of a 2-year follow-up in 94 patients, mean 79.4 ± 2.5% decrease in size of thyroid nodule was noted and all patients have compressive symptoms improved [23]. Similarly, Lim et al. reported a mean volume reduction of 93.4 ± 11.7% in a series of 111 patients undergoing RFA with a 4-year follow-up [56]. However, the reported series of laser ablation usually included patients with larger thyroid nodule and the series of RFA included patients with a higher cyst component. Thus, these results were difficult to compare. Further study to differentiate the superiority of either modality would be needed.

## 8. Conclusion

Nowadays, available reports of application of RFA in benign thyroid nodules were mainly from two centers. The main weaknesses of using RFA on the thyroid gland are several folds and they include the lack of definitive histology, possibility of incomplete nodule ablation, and surveillance problems for the residual thyroid mass after RFA. However, RFA appears to be an effective nonsurgical option to improve pressure and toxic symptoms in biopsy-proven benign thyroid nodules. Preliminary reports showed satisfactory results in volume reduction, pressure symptoms, and cosmetic symptoms, and these results appear to be sustained in the long term. Therefore, proper selection of patient with benign nodule for RFA and subsequent monitoring were needed. Further studies were needed to support its routine use in thyroid nodule.

## Figures and Tables

**Table 1:** Result of volume reduction in patients who underwent radiofrequency ablation for cold thyroid nodule.

	Number of nodule/patients	Solid component	Follow-up duration (months)	Electrode type	Number of session (mean)	Mean initial volume (mL)	Volume reduction at the 1st month (%)	Volume reduction at the last follow-up (%)
Sung et al. [11]	21/21	<10%	1–6	Internally cooled	1–3 (1.67)	10.2		92
Deandrea et al. [22]	10/9	>30%	6	Multitined expandable	1	38.7	31.7	46.3
Spiezia et al. [23]	66/66	>30%	12–24	Multitined expandable	1–3 (1.4)	21.1	43.7	76.6
Lee et al. [52]	27/27	10–50%	6–38	Internally cooled	1–4 (1.6)	14		97
Jang et al. [19]	20/20	<50%	6–43	Internally cooled	1-2 (1.1)	11.3		92
Baek et al. [17]	15/15	>50%	6–8	Internally cooled	1	7.5	49	80
Ha et al. [5]	14/11	>50%	7–92	Internally cooled		9.7		87.2
Huh et al. [53]	15/15 15/15	>50%	6	Internally cooled	1 2	13.3 13.0	40 42.7	70.2 78.3
Faggiano et al. [24]	10/10	>70%	12	Multitined expandable	1	13.3	36.5	84.9
Kim et al. [36]	35/30	0–100%	1–18	Internally cooled	1	6.3	47	64
Jeong et al. [15]	302/236	0–100%	1–41	Internally cooled	1–6 (1.4)	6.1	58	84
Lim et al. [20]	126/111	0–100%	36–81	Internally cooed	1–7 (2.2)	9.8		93.4

**Table 2:** Result of patients who underwent radiofrequency ablation for hyperfunctioning thyroid nodule.

	Number of nodule/patients	Follow-up duration (months)	Initial volume (mL)	Stopped antithyroid medication	Decreased antithyroid medication	Initial dosage of antithyroid medication (mg/d)	Dosage at last follow-up (mg/d)
Deandrea et al. [22]	23/22	6	22.5	22.7%	77.3%	5.1	2.6*
Spiezia et al. [23]	28/28	12–24	32.7	Pretoxic: 100% Toxic: 53%		7.9	4.2*
Baek et al. [16]	9/9	6–17	15	88.9%			
Faggiano et al. [24]	10/10	12	13.3	40%	40%	6.5	2.3*

*P < 0.05.
